# Evaluating the effectiveness of evidence-based falls prevention programs: a study on participant risk levels and program congruency

**DOI:** 10.3389/fpubh.2025.1517322

**Published:** 2025-02-13

**Authors:** Cathy S. Elrod, Rita A. Wong

**Affiliations:** Center for Optimal Aging, Marymount University, Arlington, VA, United States

**Keywords:** older adults, evidence-based programs, fall prevention, fall risk, health promotion, program evaluation, United States

## Abstract

**Background:**

Falls are a leading cause of injury and injury-related deaths in older adults. A variety of community-delivered, evidence-based, fall risk-reduction programs have been developed and proven effective. These evidence-based fall prevention programs (EBFPP) have been classified along a fall-risk continuum, indicating the target fall-risk level of participants. The congruency between the program’s targeted and enrolled fall-risk level of participants is unknown. This study creates a fall-risk classification index, places participants into one of three fall risk categories, and then examines congruency of actual vs. recommended fall-risk of participants, by program.

**Methods:**

Data came from the Healthy Aging Programs Integrated Database, created by the National Council on Aging (NCOA) funded by the Administration for Community Living (ACL) for use by ACL falls prevention program grantees. Using data from a pre-participation survey designed by the ACDL for their grantees, a fall risk index was created. The fall risk levels of the participants were then compared to the fall risk profile of the EBFPPs as identified in NCOA’s Evidence-based Falls Prevention Programs Risk Continuum Guidance for Program Selection in which they were enrolled.

**Results:**

Between July 2016 and June 2022, 105,323 older adults participated in one of eight EBFPPs. Participant characteristics varied among programs. Applying the fall risk index to the fall risk sample (31,064 older adults), 29% of participants were identified as being at high risk, 41% at moderate risk, and 30% at low risk. When the fall risk level of participants, by program, was compared to the target risk profile of the associated EBFPP, programs that had a risk profile targeting individuals at moderate to low risk were found to enroll a larger percentage of adults at high risk than expected. All programs enrolled at least some participants at each of the three risk levels.

**Conclusion:**

All eight EBFPPs enrolled participants across all three fall-risk levels with most programs being at least somewhat congruent with the fall-risk program continuum recommendations. More research is needed to better understand inconsistencies between risk-levels of program, target risk-levels, and actual participant risk-level, to guide either adaptations in the risk-level classification or program modifications to accommodate different risk-levels.

## 1 Introduction

Falls are a significant public health issue for older adults, impacting morbidity and mortality. They are the leading cause of injury and injury-related deaths of older adults. Indeed, although the overall mortality rate in the United States decreased between 1999 and 2020, the death rate from falls increased (1). Fear of falling and sequalae of fall injuries affect quality of life and frequently lead to lowered physical activity and fitness as well as increased risk of future falls (2). Many conditions can contribute to falls, and most falls are caused by a combination of factors (2–4). As the number of risk factors increase, the likelihood of an older adult experiencing a fall also increases.

Well established risk-reduction programs, proven to decrease fear of falling, number of falls, and, in some cases, injuries from falls, in community-dwelling older adults, are available as community-delivered education, self-management, and/or exercise programs (5, 6). Despite their known effectiveness, community dissemination has been challenging (7). Insufficient numbers of volunteer or staff program leaders, limited funding to deliver programs affordably, and lack of public awareness and interest in pursuing fall prevention activities are frequently identified factors impacting reach and program sustainability (5, 6).

Since 2014, to stimulate community adoption, the Administration for Community Living (ACL) within the Department of Health and Human Services, has supported the community dissemination of evidence-based falls prevention programs (EBFPPs) through discretionary funding awards made through a competitive application process. Community-delivered fall prevention programs that are eligible for support through this grant program are approved by the ACL through a rigorous effectiveness review process. Criteria for receiving these cooperative agreement grants include a comprehensive review of the community needs with identification of fall risks, target audiences, and rationale for the chosen evidence-based fall prevention program (EBFPP) (8). Over $50 million has been awarded by the ACL through discretionary grants to grantees in support of fall prevention (9).

As of June 2022, 16 fall prevention programs were approved by the ACL as meeting the evidence-based standard of effectiveness for community-dwelling older adults. Each program has unique characteristics and differences in their approach to fall prevention. They also vary in fall risk level of the participants they target, tailoring intervention strategies (e.g., health education, self-management training, exercise) accordingly. The National Falls Prevention Resource Center at the National Council on Aging (NCOA) developed a falls risk continuum and recommends that fall prevention stakeholders consider fall risk along that continuum (low to high) to offer programming that addresses need across different levels of the risk continuum (10). This framework can be used to help service providers guide community members to the EBFPP most suited to address their individual fall risk.

Older adults generally self-enroll in one of these ACL grant supported programs based on availability, access, and interest, completing a standardized pre-participation survey documenting selected demographic, health, and fall history items. Awardees submit this data to a national fall prevention database (HAPID) that serves as a central data repository for all awardees and all programs. A 2021 article by Brach et al. provides an overview of the demographic characteristics and fall history of the nearly 89,000 older adults across all fall prevention programs with data submitted to this central repository between 2014 and 2019 (6). Brach reported that, in the aggregate, a higher proportion of participants were female, white, and college-educated than the general US population of older adults. The number self-reporting a recent fall was similar to national averages (30% of those responding to the question). The Brach study did not explore differences among programs or attempt to differentiate participants along fall risk levels.

We are unaware of any investigation exploring the demographic difference among participants across various EBFPPs in this large national dataset or congruency of the fall risk level of the participant and the choice of the EBFPP into which they enrolled. Thus, the goals of this project were to determine: (1) participant characteristics based on enrollment in an EBFPP; (2) the fall risk levels of individuals participating in each EBFPP, and (3) agreement between the fall risk levels of participants in each EBFPP with the NCOA identified targeted risk levels for each EBFPP.

## 2 Materials and methods

### 2.1 Research questions

Three primary research questions guided this study:

1. Are there differences among the ACL approved EBFPPs in terms of participant self-reported demographic background and health history? If yes, which differences are most pronounced?
2. For each EBFPP, what proportion of participants fall into each fall risk category, based on a fall risk index calculated from variables available in the HAPID database?
3. How consistent is the risk level for each EBFPP as visualized in the NCOA Risk Level Continuum as compared to the risk levels calculated from responses provided by actual participants?

### 2.2 Study participants and procedures

Data for this project came from a national falls database, Healthy Aging Programs Integrated Database (HAPID), created and managed by the NCOA and funded by the Administration for Community Living. ACL-funded fall prevention program grantees are required to submit program data into HAPID to help ACL monitor grantees’ performance, describe participant demographics, and evaluate outcomes (11). Participants in these programs completed a standardized pre-participation survey documenting demographic and self-reported fall risk factors. Data were also collected about workshop leaders and organizations hosting programs. Data collection methods included paper and verbally administered questionnaires and electronic data capture. Workshop leaders, grant personnel, and organization staff entered data into the database. Marymount University’s Institutional Review Board designated the project as exempt since the data from the database were de-identified.

Marymount University received permission to analyze data entered into the database between July 2016 and June 2022. During this period, 56 grantees supported 16 EBFPPs in 37 states with a range of organizations delivering the programs. These organizations included: Area Agencies on Aging, health care organizations, multipurpose social services organizations, educational institutions, state and county health departments, recreational organizations, senior and community centers, residential facilities, and faith-based organizations.

EBFPPs with the largest number of participants were examined for this project (Table 1): a Matter of Balance (MOB), Bingocize, Enhance Fitness, Otago Exercise Program (Otago), Stay Active and Independent for Life (SAIL), Stepping On, Tai Chi for Arthritis, and Tai Ji Quan.

**Table 1:** Total number of participants in the eight targeted evidence-based fall prevention programs, and the subset of participants who responded to all fall risk index questions.

Program	All Participants *N* (%)	Participants who responded to all fall risk index questions *N*	Percentage of participants within each EBFPP who responded to all fall risk index questions
MOB	45,904 (43.6)	11,919	26%
Tai Chi for Arthritis	18,740 (17.5)	6,262	33%
Stepping On	14,117 (13.4)	4,279	30%
Enhance fitness	8,694 (8.3)	1,339	15%
Tai Ji Quan	8,578 (8.1)	2,641	31%
SAIL	6,863 (6.5)	3,268	48%
Bingocize	2,235 (2.1)	1,199	54%
Otago	462 (0.4)	157	34%
Total	105,323	31,064	30%

### 2.3 Data and measures

#### 2.3.1 Participant information

De-identified data from all participants above the age of 54 who enrolled in one of the eight targeted EBFPPs that started on or after July 1, 2016 and ended on or before June 30, 2022 were included in this study. The HAPID pre-participation survey that all participants were asked to complete at the start of each program provided the data used for the analysis. This survey includes demographic characteristics (e.g., age, sex, living arrangements, ethnicity, race, educational level, chronic conditions, and referral by a health care provider) and outcome measures (e.g., self-reported general health, fall history, fear of falling).

We anchored our risk level indicators in items supported by research and included or inferred in the Center for Disease Control and Prevention’s Stopping Elderly Accidents, Deaths, and Injuries (STEADI) risk screening algorithm (Table 2). Although many factors contribute to risk of falling, the factors chosen to categorize older adults into fall risk levels are varied and reliant on available data (12–19). The items included in the pre-participation survey were not created to specifically assess risk level. However, several items provide insight into fall risk.

**Table 2:** Creating the fall risk index: fall risk indicators from STEADI, related resources, and the HAPID pre-participation survey.

STEADI survey items from the STEADI self-assessment fall risk checklist (20, 21) with the associated fall risk index score	Comparable survey items from the ACL pre-participation survey with the associated fall risk index score
I have fallen in the past year. Yes, I have fallen = 2 points No, I have not fallen = 0 points	In the past 3 months, how many times have you fallen? Fallen ≥ 1 = 2 points Fallen 0 times = 0 points
I use or have been advised to use a cane or walker to get around safely. Yes = 2 points No = 0 points	Did your doctor, nurse, physical therapist or other health care provider suggest that you take this program? Yes = 2 points No = 0 points
I am worried about falling. Yes = 1 point No = 0 points	How fearful are you of falling? A little, Somewhat, A lot = 1 point Not at all = 0 points
I often feel sad or depressed. Yes = 1 point No = 0 points	Has a health care provider ever told you that you have any of the following chronic conditions that lasted 3 months or more? For the health condition of Depression: Yes = 1 point No = 0 points
Literature supported	
Moreland et al. (18) found as self-reported health declined from excellent to poor, the percentage of falls increased.	In general, would you say that your health is _____ (poor to excellent)? Fair or poor = 1 point Excellent, very good, or good = 0 points
Although everyone over 65 years of age is at somewhat increased risk (thus the entire sample), Helsel et al. (19) identified 75–84 years of age as the period of highest risk of falls.	Age of participant 75–84 years of age = 1 point <75 or > 84 years of age = 0 points

**Figure 1:**
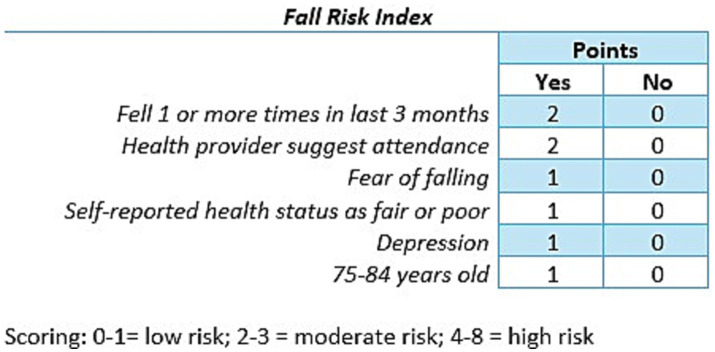
Fall risk index.

Given the information available on the pre-participation survey, we identified survey items associated with known fall risk factors to create an index to estimate fall risk level of participants at entry into the program. Six factors were identified and used to create a risk index (Figure 1). These factors included (1) falling in the last 3 months, (2) referred by a health care provider, (3) fear of falling, (4) fair or poor self-reported health status, (5) depression, and (6) age 75–84 years.

The STEADI initiative provides tools for older adults, caregivers and health care providers to reduce fall risk in older adults. The STEADI Algorithm for Fall Risk Screening, Assessment, and Intervention categorizes individuals into low, moderate or high risk for falls (20). It aligns with clinical practice guidelines, has been validated in adults aged 65 and older who participated in the National Health and Aging Trends Study, and has fair predictive validity (12–14). Lohman et al. (13) found STEADI fall risk categories were strong predictors of future falls. Individuals categorized as moderate risk were 2.6 times more likely to experience a future fall, and individuals categorized as high risk were 4.7 times as likely to experience a future fall as compared to individuals at low risk. The algorithm uses the STEADI Stay Independent checklist within the STEADI Stay Independent: Learn More about Fall Prevention brochure to determine an individual’s risk for falling (20). The STEADI fall risk self-assessment tool was developed for adults 65 years or older who are ambulatory and living within the community. Its intended use is screening for fall risk, leading to awareness of an individual’s own risk level and conversations about strategies to decrease risk (21). Rubenstein et al. compared the self-reported scores to a clinical assessment by geriatricians and found that the final 12 item questionnaire had good concurrent validity (21).

Two of the statements within the STEADI checklist assign 2 points for a yes answer, indicating a higher risk, when compared to the remaining 10 statements which assign 1 point (21). These two statements ask about fall history and gait deficiency. When comparing these two statements with ACL’s pre-participation survey, two items captured similar information (Table 2). Both the STEADI question, asking if the individual had been advised to use an assistive device and the pre-participation question, asking if a health care provider recommended that they take an EBFPP, imply that a third party deemed the individual at sufficient risk of falling to recommend an active intervention. Thus, the item was included in our fall risk index with a score of 2 points if the participant answered yes (Table 2).

The remaining items in our fall risk index (fear of falling, depression, health status, and older age) received a score of 1 point. Fear of falling (22, 23) and depression (15–17, 24), have comparable STEADI questions (Table 2) and are regularly identified as risk factors. In terms of health status, the ACL survey asked participants “In general, would you say that your health is: excellent, very good, good, fair, poor.” The STEADI checklist, although not directly asking about self-report health status, includes several items that are common indicators of impaired health status. Data from 2018 Behavioral Risk Factor Surveillance System showed that in adults aged 65 and older, as self-reported health declined from excellent to poor, the percentage of falls increased from 5 to 25% (18). Thus, a response of “fair or poor” health status received a score of 1 point.

In terms of age, Helsel et al. (19) reported that age of 75–84 years were significant predictors of falls over 4 years in community dwelling older adults. They also found that respondents over the age of 85 did not have an increased risk for falls as compared to those less than 70 years old. They hypothesized that decreased mobility and fewer risky activities could lead to a lower risk profile in individuals over 85 years of age. Thus, participants 75–84 years of age were assigned 1 point in the fall risk index.

#### 2.3.2 Fall risk categories

The following categories were used for our fall risk index: low risk = 0–1 point, moderate risk = 2–3 points, high risk = 4 or more points. We based our categories on the STEADI risk algorithm which identified individuals as low, moderate, or high risk (20). For STEADI, individuals were categorized as low risk if they scored <4/14 on the Stay Independent questionnaire or indicated ‘no’ for their fall history. In order to capture a similar categorization, we identified low risk as scoring 0–1 points. For STEADI, a score of ≥4/14 or having a fall history puts an individual automatically into moderate or high risk with high risk reserved for individuals who have gait, strength or balance problems, ≥2 falls, or at least one fall resulting in injury. Given the limited questions on the pre-participation survey, it was not possible to mimic the STEADI risk classification for moderate and high risk. Thus, we reviewed the American Geriatrics Society/British Geriatrics Society (AGS/BGS) guidelines as many algorithms are based on them (25, 26). The screening questions are like those used by STEADI and support that as the number of fall risk factors increase, fall risk increases. We chose 2–3 points as moderate risk as a participant could have one significant risk factor (2 points) or 2–3 risk factors that were consistent with STEADI items that had a score of 1 point. If a participant scored 4 points or greater, indicating more items were scored as present, the risk level was deemed high.

#### 2.3.3 NCOA’s EBFPP risk continuum

**Figure 2:**
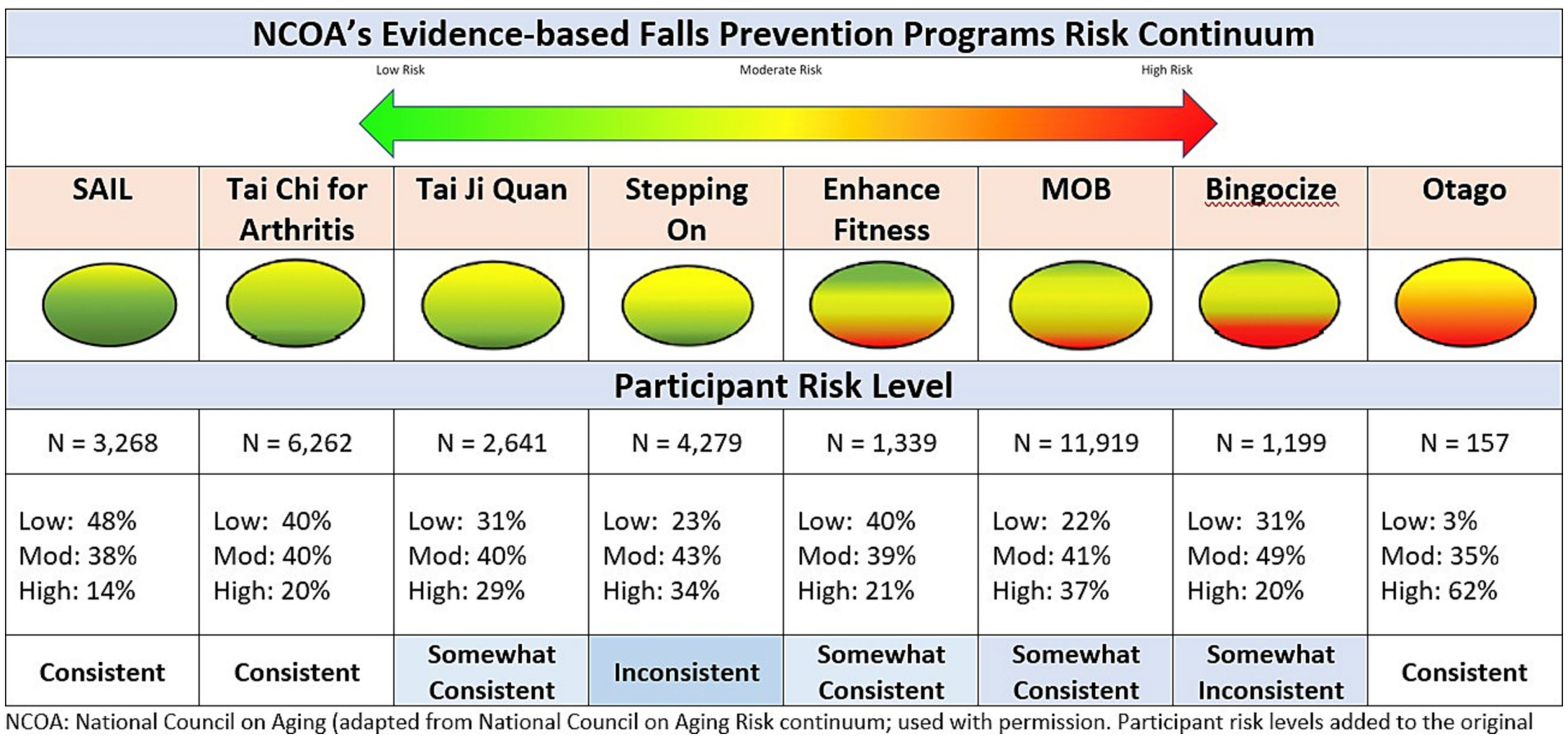
Consistency of participant risk level and programs risk continuum.

The National Falls Prevention Resource Center at NCOA developed the Evidence-based Falls Prevention Programs Risk Continuum Guidance for Program Selection as a resource for stakeholders deciding which fall prevention program to implement (10). It provides the risk level associated with the targeted population for each approved EBFPP along a continuum from low risk to high risk (Figure 2).

### 2.4 Statistical analysis

Descriptive statistics (frequencies, percentages) were used to capture participant characteristics. SPSS version 29 (IBM SPSS Statistics) was used for statistical analysis.

## 3 Results

### 3.1 Participant characteristics

#### 3.1.1 Total sample

There were 105,323 participants within the 8 EBFPPs that either started or ended a workshop between July 1, 2016 and June 30, 2022. As the database does not follow a unique individual across or within programs, and all data available to us were fully de-identified, the same individual could be in the database more than once although that individual would only be included as a single participant within a specific workshop. The top five states in which these programs were located were Minnesota (12.4%), North Carolina (8.6%), Wisconsin (6.7%), Massachusetts (6.2%), and New York (5.9%). Thirteen states (Alaska, Delaware, Hawaii, Idaho, Indiana, Kansas, Kentucky, Louisiana, Nebraska, Nevada, Oregon, Pennsylvania, and West Virginia) did not host any programs.

Characteristics of participants within the total sample are shown in Table 3. Three quarters of the individuals were between the ages of 65 and 84 years old. Eighty-two percent (82%) were female, 85% were White, 95% were not Hispanic, 45% lived alone, and 43% were a college graduate. Overall, they self-reported good, very good, or excellent as their health status (84%), with 49% self-reporting they had arthritis, heart disease (19%), diabetes (18%), depression (13%), glaucoma (12%), and lung disease (7%). Twenty-seven percent (27%) had at least one fall in the last 3 months and 85% were fearful of falling.

**Table 3:** Baseline characteristics of participants within the total sample; and those who completed all six fall risk index questions compared to those who did not.

Baseline characteristic	Total sample *n* = 105,323	Completed all 6 fall risk index questions *n* = 31,064	Did not complete all 6 fall risk index questions *n* = 74,259	
	Mean +/− SD or %	Mean +/− SD or %	Mean +/− SD or %	*p* value
Age, y	75.5 +/− 8.3	74.6 +/− 8.2	75.9 +/− 8.3	<0.001
55–64	9.1	10.6	13.5	
65–74	37.6	40.8	36.3	
75–84	38.0	36.0	38.9	
85 and above	15.3	12.6	16.4	
Sex	101,859	30,302	71,557	0.011
Female	81.9	82.3	81.7	
Lives alone	98,541	30,265	68,276	0.281
Yes	45.0	44.8	45.2	
Hispanic	96,901	30,002	66,899	<0.001
Yes	4.9	3.7	5.5	
Race	98,415	30,035	68,380	<0.001
American Indian or Alaska Native	1.2	0.7	1.4	
Asian	3.8	2.7	4.2	
Black or African American	8.3	9.1	8.0	
Native Hawaiian or Pacific Islander	0.1	0.1	0.1	
White	85.7	86.3	85.5	
More than one race	0.9	1.1	0.8	
Education	95,357	29,993	65,364	<0.001
Some elementary, middle, or high school	6.4	5.8	6.7	
High school graduate or GED	21.0	19.6	21.6	
Some college or technical school	29.7	29.8	29.7	
College (4 years or more)	42.9	44.8	42.0	
General health	89,266	31,064	58,202	<0.001
Excellent or very good	38.6	37.3	39.2	
Good	45.8	45.0	46.2	
Fair or poor	15.6	17.3	14.6	
Chronic conditions	105,323	31,064	74,259	
Arthritis	48.7	59.5	44.2	<0.001
Breathing/lung disease	7.1	12.6	4.7	<0.001
Depression	28.8	32.3	5.2	<0.001
Diabetes	17.8	21.2	16.4	<0.001
Glaucoma	31.2	13.8	11.4	<0.001
Heart disease	19.3	22.0	18.1	<0.001
Fall history	76,690	31,064	45,626	<0.001
At least one fall in last 3 months	27.3	28.4	26.6	
How fearful of falling	88,368	31,064	57,304	<0.001
Not at all	14.9	15.4	14.7	
A little	39.0	38.1	39.5	
Somewhat	32.6	31.8	33.0	
A lot	13.5	14.7	12.9	
Referred by health care provider	81,948	31,064	50,884	
Yes	16.4	16.7	16.1	0.016

When comparing participant characteristics across all EBFPPs there were differences based on enrollment in a specific program (Table 4).

- Bingocize participants were more likely to be non-White (63%), Hispanic (16.8%), living alone (48%), no more than a high school graduate (48.1%), in fair or poor health (24.4%), and not fearful of falling (26%).
- Enhance fitness participants were more likely to be a high school graduate with at least some college or technical school education (84%), referred by a health care professional (24.1%), and not fearful of falling (22.5%).
- Matter of balance participants were more likely to be living alone (50.1%), depressed (34.7%), a previous faller (32.45%), and fearful of falling (89%).
- Otago exercise program participants were more likely to be male (26.35%), at least 85 years or older, graduated college (56.9%), in fair or poor health (23.8%), previous faller (40.8%), and referred by a health care professional (45.1%).
- Stay Active and Independent for Life participants were more likely to be female (87.6%), in excellent or very good health (50.5%), and not fearful of falling (25.7%).
- Stepping On participants were more likely to be living alone (46.9%), previous faller (33.3%), and fearful of falling (92.3%).
- Tai Chi Arthritis participants were more likely to be in excellent or very good health (46.85%) and not fearful of falling (22.2%).
- Tai Ji Quan participants did not trend toward any unique characteristics when compared to the general participant profile.

**Table 4:** Characteristics of participants in total sample.

	Total Sample	Bingocize	Enhance Fitness	MOB	Otago	SAIL	Stepping On	Tai Chi Arthritis	Tai Ji Quan
	*n* (%)	*n* (%)	*n* (%)	*n* (%)	*n* (%)	*n* (%)	*n* (%)	*n* (%)	*n* (%)
	105,323	2,235	8,694	45,904	462	6,863	14,117	18,470	8,578
Age	105,323	2,235	8,694	45,904	462	6,863	14,117	18,470	8,578
55–64	9,560 (9.1)	322 (14.4)	845 (9.7)	3,249 (7.1)	32 (6.9)	548 (8.0)	917 (5.8)	2,628 (14.2)	1,119 (13.0)
65–74	39,614 (37.6)	878 (39.3)	3,956 (45.5)	14,793 (32.2)	127 (27.5)	2,966 (43.2)	4,376 (31.0)	8,723 (47.2)	3,795 (44.2)
75–84	40,050 (38.0)	740 (33.1)	3,209 (36.9)	18,752 (40.9)	185 (40.0)	2,638 (38.4)	6,168 (43.7)	5,641 (30.5)	2,717 (31.7)
85 and above	16,099 (15.3)	295 (13.2)	684 (7.9)	9,110 (19.8)	118 (25.5)	711 (10.4)	2,756 (19.5)	1,478 (8.0)	947 (11.0)
Sex	101,859	2,000	8,293	44,721	452	6,176	13,746	18,146	8,325
Male	18,486 (18.1)	394 (19.7)	1,337 (16.1)	8,637 (19.3)	119 (26.3)	763 (12.4)	2,788 (20.3)	2,929 (16.1)	1,519 (18.2)
Female	83,373 (81.9)	1,606 (80.3)	6,956 (83.9)	36,084 (80.7)	333 (73.7)	5,413 (87.6)	10,958 (79.7)	15,217 (83.9)	6,806 (81.8)
Living alone	98,541	2,124	7,570	43,816	370	6,403	12,867	17,306	8,085
No	54,157 (55.0)	1,104 (52.0)	4,666 (61.6)	21,883 (49.9)	218 (58.9)	3,955 (61.8)	6,833 (53.1)	10,738 (62.0)	4,760 (58.9)
Yes	44,384 (45.0)	1,020 (48.0)	2,904 (38.4)	21,933 (50.1)	152 (41.1)	2,448 (38.2)	6,034 (46.9)	6,568 (38.0)	3,325 (41.1)
Hispanic	96,901	1.928	7,311	43,275	355	5,646	13,356	17,359	7.671
No	92,150 (95.1)	1,605 (83.2)	6,932 (94.8)	40,608 (93.8)	339 (95.5)	5,508 (97.6)	13,165 (98.6)	16,596 (95.6)	7,397 (96.4)
Yes	4,751 (4.9)	323 (16.8)	379 (5.2)	2,667 (6.2)	16 (4.5)	138 (2.4)	191 (1.4)	763 (4.4)	274 (3.6)
Race	98,415	1,952	7,169	43,476	424	6,315	13,415	17,433	8,231
White	84,368 (85.7)	1,230 (63.0)	6,476 (90.3)	36,292 (83.5)	375 (88.4)	5,559 (88.0)	12,318 (91.8)	15,145 (86.9)	6,974 (84.7)
Black or African American	8,205 (8.3)	601 (30.8)	453 (6.3)	4,457 (10.3)	37 (8.7)	330 (5.2)	695 (5.2)	1,305 (7.5)	327 (4.0)
Asian	3,691 (3.8)	81 (4.1)	211 (2.9)	1,786 (4.1)	5 (1.2)	327 (5.2)	172 (1.3)	521 (3.0)	588 (7.1)
American Indian or Alaska Native	1,149 (1.2)	16 (0.8)	19 (0.3)	490 (1.1)	2 (0.5)	31 (0.5)	154 (1.1)	230 (1.3)	207 (2.5)
Native Hawaiian or other Pacific Islander	117 (0.1)	7 (0.4)	11 (0.2)	49 (0.1)	0 (0.0)	7 (0.1)	8 (0.1)	29 (0.2)	6 (0.1)
More than one race	885 (0.9)	17 (0.9)	0.0 (0)	402 (0.9)	5 (1.2)	1.0 (61)	68 (0.5)	203 (1.2)	129 (1.6)
Education level	95,357	1,961	7,438	40,452	422	5,948	13,514	17,572	8,050
Some elementary, middle, or high school	6,104 (6.4)	377 (19.2)	87 (1.2)	3,732 (9.2)	17 (4.0)	212 (3.6)	534 (4.0)	524 (3.0)	621 (7.7)
High school graduate or GED	19,995 (21.0)	567 (28.9)	1,102 (14.8)	9,609 (23.8)	68 (16.1)	1,046 (17.6)	3,652 (27.0)	2,738 (15.6)	1,213 (15.1)
Some college or technical school	28,344 (29.7)	545 (27.8)	2,405 (32.3)	12,235 (30.2)	97 (23.0)	1,756 (29.5)	4,169 (30.8)	4,960 (28.2)	2,177 (27.0)
College (4 years or more)	40,914 (42.9)	472 (24.1)	3,844 (51.7)	14,876 (36.8)	240 (56.9)	2,934 (49.3)	5,159 (38.2)	9,350 (53.2)	4,039 (50.2)
General health	89,266	2,044	2,023	39,852	411	6,226	13,144	17,476	8,090
Excellent or very good	34,501 (38.6)	566 (27.7)	801 (39.6)	13,441 (33.7)	129 (31.4)	3,145 (50.5)	4,701 (35.8)	8,175 (46.8)	3,543 (43.8)
Good	40,872 (45.8)	981 (48.0)	897 (44.3)	18,917 (47.5)	184 (44.8)	2,552 (41.0)	6,435 (49.0)	7,540 (43.1)	3,366 (41.6)
Fair or poor	13,893 (15.6)	497 (24.3)	325 (16.1)	7,494 (18.8)	98 (23.8)	529 (8.5)	2,008 (15.3)	1,761 (10.1)	1,181 (14.6)
Chronic conditions	105,323	2,235	8,694	45,904	462	6,863	14,117	18,470	8,578
Arthritis	51,269 (48.7)	968 (43.3)	2,104 (24.2)	24,041 (24.2)	226 (48.9)	2,595 (37.8)	7,177 (50.8)	10,403 (56.3)	3,755 (43.8)
Breathing/lung disease	7,447 (7.1)	339 (15.2)	500 (5.8)	2,749 (6.0)	49 (10.6)	726 (10.6)	994 (7.0)	1,398 (7.6)	692 (8.1)
Depression	13,895 (13.2)	386 (17.3)	615 (7.1)	6,600 (14.4)	78 (16.9)	726 (10.6)	1,969 (13.9)	2,318 (12.6)	1,212 (14.1)
Diabetes	18,717 (17.8)	616 (27.6)	711 (8.2)	9,544 (20.8)	81 (17.5)	878 (12.8)	2,706 (19.2)	2,834 (15.3)	1,347 (15.7)
Glaucoma	12,724 (12.1)	109 (4.9)	466 (5.4)	6,813 (14.8)	80 (17.3)	915 (13.3)	1,730 (12.3)	1,802 (9.8)	809 (9.4)
Heart disease	20,296 (19.3)	396 (17.7)	674 (7.8)	10,425 (22.7)	114 (24.7)	930 (13.6)	2,985 (21.1)	3,401 (18.4)	1,371 (16.0)
Fall history	76,690	1,699	1,974	34,095	387	5,430	11,905	14,485	6,715
No	55,728 (72.7)	1,379 (81.2)	1,586 (80.3)	23,059 (67.6)	229 (59.2)	4,611 (84.9)	7,941 (66.7)	11,718 (80.9)	5,205 (77.5)
At least one fall in last 3 months	20,962 (27.3)	320 (18.8)	388 (19.7)	11,036 (32.4)	158 (40.8)	819 (15.1)	3,964 (33.3)	2,767 (19.1)	1,510 (22.5)
How fearful of falling	88,368	1,961	1,824	39,567	349	6,092	13,147	17,377	8,051
Not at all	13,176 (14.9)	509 (26.0)	410 (22.5)	4,338 (11.0)	46 (13.2)	1,565 (25.7)	1,008 (7.7)	3,866 (22.2)	1,434 (17.8)
A little	34,456 (39.0)	693 (35.3)	742 (40.7)	14,380 (36.3)	105 (30.1)	2,845 (46.7)	4,558 (34.7)	7,550 (43.4)	3,583 (44.5)
Somewhat	28,793 (32.6)	520 (26.5)	509 (27.9)	14,391 (36.4)	129 (37.0)	1,364 (22.4)	5,075 (38.6)	4,644 (26.7)	2,161 (26.8)
A lot	11,943 (13.5)	239 (12.2)	163 (8.9)	6,458 (16.3)	69 (19.8)	318 (5.2)	2,506 (19.1)	1,317 (7.6)	873 (10.8)
Referral	81,948	1,833	3,674	37,187	306	4,829	12,401	15,558	6,160
No	68,548 (83.6)	1,542 (84.1)	2,788 (75.9)	31,107 (83.7)	168 (54.9)	4,179 (86.5)	10,701 (86.3)	13,086 (84.1)	4,977 (80.8)
Yes	13,400 (16.4)	291 (15.9)	886 (24.1)	6,080 (16.3)	138 (45.1)	650 (13.5)	1,700 (13.7)	2,472 (15.9)	1,183 (19.2)

#### 3.1.2 Fall risk sample

Of the 105,323 participants in the dataset, 31,064 answered all six pre-participation questions that made up the fall risk index and, thus, were included in the calculations of fall risk levels. All other participants were excluded from the calculation of risk level. Table 3 displays the response by group (those included vs. those excluded from calculation of fall risk index) and Chi Square analyses for group differences. A *p* < 0.01 level of significance was chosen given the large number of variables that increase the risk of a Type I error. Statistically significant differences were reported for all variables except, sex, living situation, and referral from a healthcare provider. Demonstrating statistically significant differences was not surprising given the large dataset. As seen in the table, for most variables, the actual differences were small and unlikely to represent clinically important differences.

Notable differences include more individuals with poor to fair health in the inclusion group and fewer in excellent to good health as well as more individuals in every category of chronic condition in the inclusion group than the exclusion group. This was particularly evident for depression with large differences between the groups (32.3% vs. 13.2%). Differences in those reporting arthritis (60% vs. 44%) and breathing/lung issues (13% vs. 5%) are also notable.

#### 3.1.3 Fall risk level

**Figure 3:**
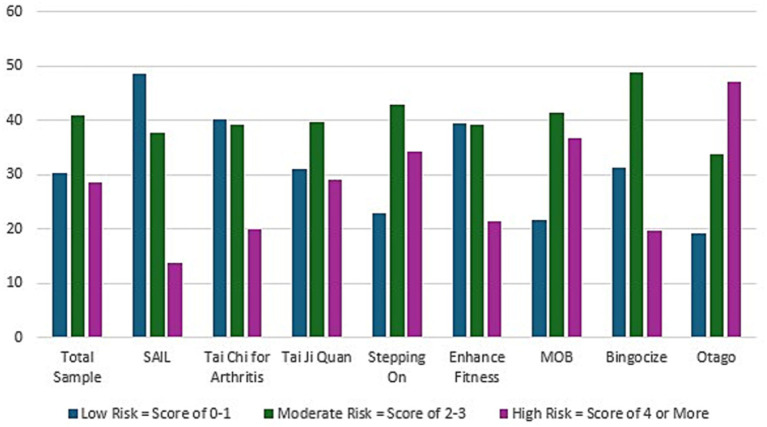
Fall risk level of participants by evidence-based falls prevention program.

Using the fall risk index within the fall risk sample (31,064 older adults), 29% of participants were identified as being at high risk, 41% at moderate risk, and 30% at low risk. Figure 3 provides the breakdown of the fall risk level of participants by each evidence-based fall prevention program. Stay Active and Independent for Life (SAIL) had the largest percentage of individuals at low risk (48.5%) and Otago had the lowest (19%). Whereas, Otago had the largest percentage at high risk (47%) and SAIL with the lowest (14%).

#### 3.2 Congruency of targeted risk profiles

Congruency was examined between the targeted risk levels of participants as depicted on the NCOA visual continuum and the risk levels derived from our fall risk index. In general, greater congruency was observed in programs at either end of the continuum (high or low ends) than midrange. As depicted in Figure 2, the NCOA fall risk continuum visually illustrates each EBFPP’s targeted participant risk level (green = low risk, yellow = medium risk, red = high risk). For each program targeting only two of the three fall risk categories, we operationally defined ‘Consistent’ as having at least 80% of participants classified within the target risk level, ‘Somewhat Consistent’ if between 70 and 79% of participants were classified within the two risk categories, and ‘Inconsistent’ if less than 70% of participants were classified within the two risk levels. For programs targeting participants across all three risk levels, we examined differences between the visually estimated relative proportions in each category and the proportions calculated using the fall risk index.

Five of the eight EBFPPs indicated the program targets only two of the three possible risk categories. Four programs (SAIL, Tai Chi for Arthritis, Tai Ji Quan, Stepping On) targeted only low or moderate risk individuals and one program (Otago) targeted only moderate to high-risk participants. SAIL, Tai Chi for arthritis and Otago meet the 80% criteria and were classified as ‘Consistent’. Tai Ji Quan, with 71% of participants in the target risk levels was classified as ‘Somewhat Consistent’. Stepping On with only 66% of participants within the target risk levels, was classified as ‘Inconsistent’.

Three EBFPPs (Enhance fitness, MOB, Bingocize) included all three risk levels in their target population with slightly different proportions visually depicted for each along the NCOA continuum. All three enrolled participants across all three risk levels. All three were classified as ‘Somewhat Consistent’ given variations from the NCOA visual depiction. Enhance Fitness and Bingocize, visually depicted as targeting participants fairly equally across all three levels, attracted fewer participants at high risk than visually illustrated. MOB, depicted as targeting predominantly moderate and low risk participants, enrolled a high proportion of high-risk participants.

## 4 Discussion

Using data from the ACL-NCOA Healthy Aging Programs Integrated Database (HAPID) between July 2016 and June 2022, we examined the characteristics of a broad national sample of older adults attending a variety of evidence-based fall prevention programs (EBFPPs) offered by a range of older adult-community serving agencies within 37 of the 50 states within the United States. The characteristics of the 105,323 participants displayed in Table 4 indicate that participants were primarily: white; female; with at least some fear of falling; between 65 and 84 years of age; had completed at least some college; described their health as good, very good, or excellent; and self-enrolled in the EBFPP (were NOT referred to the program by a health care provider). These findings are similar to the demographic trends identified in earlier studies by Smith et al., based on 2014–2017 HAPID data and Brach, based on 2014–2019 HAPID data (6, 27). Of particular concern is the continued low enrollment of men and participants who are non-white.

We used data available in the pre-participation survey inputted into the national HAPID database to create a fall risk score for each participant in the eight ACL-supported fall prevention programs and categorized risk into three levels. As such, 29% of participants were classified at high fall risk, 41% at moderate fall risk, and 30% at low fall risk. The proportion of individuals at moderate to high risk (70%) was higher than the 48% of individuals identified as moderate or high risk in the 2011 National Health and Aging Trends Study (NHATS) of the general older adult population (13). Additionally, 85% of subjects in our sample expressed at least some fear of falling compared to only 25.6% of subjects in the 2011 NHATS survey (19). These findings support the assumption that individuals at higher fall risk and with at least some concern about falling are more likely to enroll in a fall prevention program than those at low risk and low concern about falling.

In creating the fall risk index, we were limited to the variables included in the pre-participation survey and reported in the HAPID dataset. This fall risk index is not intended for generalization beyond the HAPID dataset. The CDC STEADI checklist was chosen as the primary tool against which to base risk level. The STEADI fall risk checklist was specifically designed for community-dwelling older adults, to be understandable to older adults, and to include modifiable fall risk factors. Rubenstein et al. all reported high consistency between self-reported STEADI checklist scores and clinically assessed scores (21). Future studies are needed to examine concurrent validity of responses on this fall risk index against the comparable items on the STEADI checklist and criterion-related validity by assessing the consistency of the self-reported responses with clinician assessment.

When examining each EBFPP separately (Figure 2), we see that each program enrolled participants across all three risk levels with most programs being at least somewhat consistent with their targeted level along the NCOA risk continuum. There was more congruence for programs tailored for participants at the ends of the risk level (low risk or high risk). Two programs (Stepping On and Matter of Balance), that focused more on adult education and encouraging behavior change than on active exercise participation, had very similar risk profiles with a tendency toward enrolling more participants at high-risk levels than other programs, except Otago. Stepping On, Otago, and Matter of Balance each demonstrated a higher percentage of participants who had fallen in the past 3 months (33.3, 40.8, 32.4%, respectively) and had the highest number of subjects expressing at least a little concern about falling (92, 87, 89%, respectively) than the other programs. This is consistent with the finding that these three programs enrolled the most subjects in the high-risk category.

Both Matter of Balance (MOB) and Stepping On are workshop-based programs focusing primarily on adult education, self-efficacy, and behavior change rather than on physical exercise. We speculate that older adults at higher fall risk may gravitate more toward workshop-based programs than exercise and physical activity programs. For MOB, this risk profile was somewhat consistent with their target population along the risk continuum. However, participants in Stepping On did not reflect the risk profile in NCOA’s risk continuum that identifies the program as more suitable for low-risk individuals. Future investigation of outcomes for Stepping On by risk level may help determine if the NCOA risk continuum for Stepping On should be adjusted or if high-risk participants should be recommended to an alternative program tailored more directly for high fall-risk. Otago, an exercise program targeting high-risk individuals, is unique in that it is typically, although not exclusively, led by a health professional and delivered in an individualized 1–1 format.

Interestingly, Bingocize, the only other EBFPP in our review that is primarily workshop focused, had a relatively low proportion of participants in the high-risk category despite high-risk being a substantial target group according to the NCOA risk continuum. Bingocize also had the highest proportion of participants who were non-white (37%), and had diabetes (27.6%) or breathing/lung disease (15.2%). Bingocize and Otago had the highest proportion of participants defining their overall health as ‘fair or poor’. Combining the benefit of a familiar and popular game, bingo, with adult health education, seems to capture interest across a broad fall risk level and wider-range of demographics than other EBFPPs. Future examination, by risk level, of the ability to achieve positive behavior change across risk levels will help determine the ideal risk continuum target for this program.

All EBFPPs with a primary focus on physical exercise participation (Tai Chi for Arthritis, Tai Ji Quan, Enhance Fitness, and SAIL) had fewer individuals at high risk than those enrolled in workshop-based programs (MOB and Stepping On). This was generally consistent with expectations as visualized along the NCOA risk continuum.

Evidence-based falls prevention programs have different ways of addressing fall prevention risk factors and different attractors for participants. Although subjects self-enroll in programs, program availability varies widely. Most communities are unable to offer a range of fall prevention programs. Thus, participants seeking fall prevention programs are limited to the programs available in their community even if the program is not ideally suited to their risk level. Host organizations must consider their mission, target audience, location of classes, and needs of their community in determining which programs to offer. Leaders delivering programs must be sensitive to the range of participant risk levels and strategies to adapt the program while remaining within the program’s fidelity requirements.

Limitations of this study include the limited availability of risk-factor indicators for calculation of risk level, the low number of participants completing all 6 risk-factor indicators thus included in risk factor calculation, and the non-random nature of subjects enrolled in the programs. Only 30% of subjects in the HAPID database completed all 6 questions contributing to the fall risk index, required to be included in the fall-risk level categorization. Thus, our fall risk index represents a ‘best-available’ estimate of risk level from the subset of program participants who completed all 6 risk factor questions. A comparison of key characteristics of those included vs. not-included in the risk level calculations illustrated small but statistically significant differences between the two groups on most variables which could have impacted the risk-level scores.

The sample population for the HAPID dataset was not randomly chosen from the general population of older adults seeking fall prevention programs. All programs were supported by an ACL grant. Grantees were chosen based on a highly competitive application process in which they provided compelling evidence for the need in their community, the target region, delivery processes, and participants. Examining variability by target geographic area, type of delivery, or target populations is beyond the scope of this study. However, future examinations of these factors could provide additional insights into the link between choice of programs, risk level of participants, and characteristics of attendees.

## 5 Conclusion

The community-delivered EBFPPs supported by grants from ACL have enrolled a wide range of older adults. At least 80% of individuals enrolling in the community-delivered EBFPPs supported by ACL were white, female, have at least some fear of falling, and identify their general health as good, very good, or excellent. Overall, the proportion of participants who had fallen at least once was similar to that found in the general population. However, this rate varies by program. Programs focused more on training and education enrolled more participants who have fallen than EBFPPs focused more on exercise interventions.

All programs enroll participants across all three risk levels with variability across programs. The risk levels of participants in most programs are at least somewhat consistent with the NCOA risk continuum that serves as a guideline for choosing programs to match the target audience in a given community. As only 3 programs enrolled participants that were in agreement with their targeted risk profile, further examination of the remaining programs’ identified risk level is indicated.

The enrollment of men and minority participants continues to be low, without substantial improvement from earlier studies using the same database. The findings suggest a need to tailor programs and improve recruitment to enhance reach to these under-represented populations. These findings also serve as a reminder that the characteristics of program participants vary across each of the three risk levels. This supports the NCOA recommendation to offer a range of programs targeting individuals at various risk levels. Program leaders must also be sensitive to the varied risk levels of their participants as it became clear in this study that not all older adults are enrolling in programs that are consistent with their fall risk profile.

It is crucial that programming to reduce fall risk is available for all older adults. The results of this study help to inform older-adult-focused organizations who wish to meet the needs of their community by recognizing populations who have had less access to EBFPPs and ensuring that the fall risk profile of the chosen EBFPP is congruent with the level of fall risk of their targeted audience.

## Data Availability

The datasets presented in this study can be found in online repositories. The names of the repository/repositories and accession number(s) can be found below: data for this research was sourced from the Healthy Aging Programs Integrated Database (HAPID), which is publicly available upon request and funded by ACL/HHS. Interested researchers must email HAPIDHelp@ncoa.org, submit a Data Use Agreement, and meet with data management at the National Council on Aging to access a data codebook and understand the appropriateness of the data for the proposed research.
